# Fat Phagocytosis Promotes Anti-Inflammatory Responses of Macrophages in a Mouse Model of Osteonecrosis

**DOI:** 10.3390/cells13141227

**Published:** 2024-07-20

**Authors:** Zhuo Deng, Harry K. W. Kim, Paula A. Hernandez, Yinshi Ren

**Affiliations:** 1 Center of Excellence in Hip, Scottish Rite for Children, Dallas, TX 75219, USAharry.kim@utsouthwestern.edu (H.K.W.K.); 2 Department of Orthopaedic Surgery, University of Texas Southwestern Medical Center, Dallas, TX 75390, USA

**Keywords:** osteonecrosis, macrophage, phagocytosis, necrotic fat, inflammation

## Abstract

Osteonecrosis (ON) of the femoral head (ONFH) is a devastating bone disease affecting over 20 million people worldwide. ONFH is caused by a disruption of the blood supply, leading to necrotic cell death and increased inflammation. Macrophages are the key cells mediating the inflammatory responses in ON. It is unclear what the dynamic phenotypes of macrophages are and what mechanisms may affect macrophage polarization and, therefore, the healing process. In our preliminary study, we found that there is an invasion of macrophages into the repair tissue during ON healing. Interestingly, in both ONFH patients and a mouse ON model, fat was co-labeled within macrophages using immunofluorescence staining, indicating the phagocytosis of fat by macrophages. To study the effects of fat phagocytosis on the macrophage phenotype, we set up an in vitro macrophage and fat co-culture system. We found that fat phagocytosis significantly decreased M1 marker expression, such as IL1β and iNOS, in macrophages, whereas the expression of the M2 marker Arg1 was significantly increased with fat phagocytosis. To investigate whether the polarization change is indeed mediated by phagocytosis, we treated the cells with Latrunculin A (LA, which inhibits actin polymerization and phagocytosis). LA supplementation significantly reversed the polarization marker gene changes induced by fat phagocytosis. To provide an unbiased transcriptional gene analysis, we submitted the RNA for bulk RNA sequencing. Differential gene expression (DGE) analysis revealed that the top upregulated genes were related to anti-inflammatory responses, while proinflammatory genes were significantly downregulated. Additionally, using pathway enrichment and network analyses (Metascape), we confirmed that gene-enriched categories related to proinflammatory responses were significantly downregulated in macrophages with fat phagocytosis. Finally, we validated the similar macrophage phenotype changes in vivo. To summarize, we discovered that fat phagocytosis occurs in both ONFH patients and an ON mouse model, which inhibits proinflammatory responses with increased anabolic gene expression in macrophages. This fat-phagocytosis-induced macrophage phenotype is consistent with the in vivo changes shown in the ON mouse model. Our study reveals a novel phagocytosis-mediated macrophage polarization mechanism in ON, which fills in our knowledge gaps of macrophage functions and provides new concepts in macrophage immunomodulation as a promising treatment for ON.

## 1. Introduction

Osteonecrosis (ON) is caused by a disruption of blood supply to the bone, resulting in ischemic cell death and the release of damage-associated molecular patterns (DAMPs), such as high-mobility group box 1 (HMGB1), s100, and inflammatory cytokines [1,2]. DAMPs inhibit osteoblast differentiation and elicit chronic inflammation of the joint, leading to excessive bone resorption [2]. The inflammatory responses are mediated by a whole spectrum of immune cells, including B cells, T cells, neutrophils, and macrophages [3].

Macrophages derive from the monocyte lineage and are the primary first-in-line cells in response to tissue damage or bacterial infections [4,5]. They carry important roles in presenting antigens, initiating inflammatory responses, and eliminating foreign objects (such as bacterial) or damaged cells (such as necrotic cell debris in ON) through a process called phagocytosis. Macrophages possess heterogeneous phenotypes and are dynamic cells that respond differently to different environments. In vitro, macrophages respond to stimuli differently (such as LPS vs. IL-4) and are characterized as an M1 (also known as classically activated) or M2 (alternatively activated) phenotype with distinct functions of promoting proinflammatory or pro-healing responses, respectively [6,7]. It remains unclear what the macrophage phenotypes are and how macrophages respond to tissue injury in ON.

Osteonecrosis is a complex bone disorder presenting with both acute and chronic inflammatory responses [1]. To better study the mechanisms and cell types that are involved in ON healing. We established a trauma-induced ON mouse model in which the popliteal vessel, as well as the medial, central, and lateral genicular vessels supplying the distal femoral epiphysis, can be identified and surgically cauterized. This ON mouse model shows consistent and full epiphysis necrotic cell death with phenotypes similar to those in human patients [8]. Interestingly, in both human ON and the ON rodent model, we observe a sharp increase in marrow adipose tissue [9,10], indicating altered lipid metabolism and skewed bone progenitor cell differentiation. Marrow fat tissue is an important source of cytokine/chemokine production, which has been shown to play a significant role in mediating inflammatory responses via macrophages in cancer [11]. In our preliminary study, we discovered a sharp increase in necrotic fat phagocytosis by macrophages during the early healing phases of ON, which may directly affect the responses of macrophages in tissue healing.

The purpose of this study is to investigate the macrophage phenotypes in ON and how phagocytosis of necrotic fat alters its phenotype. To study this question, we established an in vitro co-culture system that allows macrophages to make direct contact with necrotic fat. Gene expression will be analyzed via qPCR and bulk RNA sequencing. In vivo macrophage phenotypes were studied by immunofluorescent labeling at different stages of ON healing.

## 2. Materials and Methods

### 2.1. Human ONFH Samples

The protocol of the human sample collection was approved by the Institutional Review Board (IRB) at the University of Texas Southwestern Medical Center. The human ONFH specimen was retrieved from a 23-year-old ONFH patient who received total hip arthroplasty on the left side.

### 2.2. Animals

The mouse protocol used in this study was approved by the Institutional Animal Care and Use Committee (IACUC) at the University of Texas Southwestern Medical Center. LyM-cre (B6.129P2-Lyz^2tm1(cre)Ifo^/J) and tdTomato (B6.Cg-Gt(ROSA)26Sor^tm9(CAG-tdTomato)Hze^/J, referred to as TMT in the rest of the manuscript) mice were purchased from the Jackson Laboratory. The LysM-TMT mice were produced by crossbreeding and confirmed by genotyping. The LysM-TMT mice underwent surgical induction of ischemic osteonecrosis (ON) at the right distal femoral epiphysis at the age of 12 weeks, as previously described [8,10]. At 2 or 4 weeks after ON induction, mice were sacrificed for tissue retrieval (*n* = 3 for each time point). Both left (control) and right (ON) knee joints (distal femur and proximal tibia) were dissected for histological analysis. Wild-type C57BL/6 male mice (The Jackson Laboratory) were used to collect bone, bone marrow, and abdominal fat tissues. Only male mice were used in this study.

### 2.3. Histomorphometry

The human ONFH samples were fixed in 4% paraformaldehyde (PFA) for 3–5 days and decalcified in 14% ethylenediaminetetraacetic acid (EDTA) for 7–10 days, cryopreserved in 15% sucrose and then 30% sucrose for 3–5 days each, and embedded in OCT for frozen blocks. Similarly, the mouse knee joint samples were fixed in 4% PFA for 1 day, cryopreserved in 30% sucrose for 1 day, and embedded in OCT for frozen blocks. The frozen sections were cut into 10 µm thick slides for staining. Hematoxylin and eosin (H&E) staining and Oil Red O staining were processed following standard protocols [10,12]. Immunofluorescence (IF) staining was used to identify macrophage cell markers. Bodipy™ staining (Invitrogen, Waltham, MA, USA) was used to identify adipocytes and fat droplets [13]. The mouse CD163 anti-human antibody (Novus Biologicals, Centennial, CO, USA), rabbit F4/80 anti-mouse antibody (Cell Signaling Technology, Danvers, MA, USA), rabbit iNOS anti-mouse antibody (Abcam, Cambridge, United Kingdom), rabbit Arg1 anti-human antibody (Santa Cruz Biotechnology, Dallas, TX, USA), and mouse CD68 anti-rat antibody (AbD Serotec, Raleigh, NC, USA) were used as primary antibodies to identify exposed specific markers. The Alexa 594 IgG goat anti-mouse antibody and Alexa 488 IgG goat anti-rabbit antibody (Molecular Probes, Eugene, OR, USA) were used as the secondary antibodies to label the specific markers and display fluorescence signals. The mounting medium with DAPI (Vectashield, Vector Laboratories, Newark, CA, USA) was applied for nuclear staining. Color images were taken by an Olympus BX63F microscope (Olympus Life Science, Waltham, MA, USA), and fluorescence images were taken by an Axio Imager.M2 (Zeiss, Oberkochen, Germany) and analyzed by the ImageJ 1.54g software (NIH, USA).

### 2.4. Cell Culture

Mouse bone marrow-derived macrophages were isolated from the femur and tibia. Bone marrow cells were cultured on 150 mm Petri dishes in macrophage induction medium including RPMI complete medium (10% fetal bovine serum + 1% Penicillin/Streptomycin) supplemented with 20 ng/mL recombinant mouse M-CSF protein (R&D systems, Minneapolis, MN, USA) for 7 days. The medium was replaced with fresh medium on day 3. On day 7, mature macrophages were detached and harvested for the experiments. For the macrophage and necrotic fat co-culture system, cells were seeded on the Transwell^®^ cell culture inserts with a 0.4 µm pore polycarbonate membrane (Corning Inc., Corning, NY, USA), and the necrotic fat was added under the inserts to ensure direct contact between cells and fat on the next day (Figure 3A). Fresh bone and bone marrow from the femur and tibia were used to produce artificial necrotic bone fluid (aNBF) using the freeze–thaw method described previously [2]. In brief, a sterile femur and tibia were cut into small pieces around 1–2 mm in length. The bone pieces were collected in a sterile 15 mL conical tube for 5 freeze–thaw cycles, which entailed freezing in liquid nitrogen for 5 min, followed by thawing in a 37 °C water bath for 5 min. After the freeze–thaw cycles, PBS was added to dissolve the DAMPs released from the artificial necrotic bone at 10 µL/mg. Mouse abdominal fat tissue was collected to produce a necrotic control and fat solution. Briefly, the fat tissue was washed using fresh PBS 3–4 times and digested with a digestion solution (0.1% (*w*/*v*) collagenase type-1, 1% (*w*/*v*) BSA, and 1% (*v*/*v*) CaCl_2_ in PBS) in a 37 °C water bath for 1 h. The solution was neutralized by adding RPMI complete medium, and the freeze–thaw cycle was repeated 5 times (freezing in liquid nitrogen for 5 min, followed by thawing in 37 °C water bath for 5 min). The solution was filtered and centrifuged in the last step. The fat control medium (Con) was collected from the middle layer, and the high-fat-containing medium (Fat) was collected from the top fat layer (Figure S1) [14]. Macrophages were plated at 2 × 10^4^ cell/well on 6.5 mm inserts for staining and 1.5 × 10^6^ cell/well on 24 mm inserts for RNA collection. After overnight incubation, the control and fat media replaced the same portion of the medium in the corresponding bottom cell culture wells; aNBF at 10 µg/mL was added to the top Transwell^®^ inserts to mimic the necrotic environment (Figure 3A). Latrunculin A (LA) (Thermo Fisher Scientific, Waltham, MA, USA) is an inhibitor of actin polymerization and has been widely used to inhibit macrophage phagocytosis due to its low toxicity and the reversibility of its effect [15,16]. LA was added at a final concentration of 2 µM. Macrophages were harvested 24 h after the treatment. Cells on the 6.5 mm inserts were stained with Bodipy™ [13] and mounted with DAPI. The images were captured by a Zeiss Axiovert 200M Confocal Fluorescence Microscopy (Zeiss, Oberkochen, Germany).

### 2.5. Western Blot

The Pierce™ BCA Protein Assay Kit (Thermo Fisher Scientific) was used to measure the protein concentration of the acquired mouse aNBF samples. Western blotting was used to show the presence of DAMPs in aNBF [2]. Briefly, 40 µg of protein from each sample was loaded in a 12% polyacrylamide gel. Sodium Dodecyl Sulfate–Polyacrylamide Gel Electrophoresis (SDS-PAGE) was applied to separate proteins based on their molecular weight. The samples were then transferred to a PVDF membrane (LI-COR Biosciences, Lincoln, NE, USA). The primary antibodies HMGB1 (Abcam), Cyclophilin A (Thermo Fisher Scientific), S100A (Abcam), and β-Actin (LI-COR Biosciences) were used to identify the corresponding specific proteins on the membrane. The final signal was detected with the Odyssey Imaging System (LI-COR Biosciences, Lincoln, NE, USA) after inoculation with the IRDye secondary antibody against the primary antibody.

### 2.6. RNA Isolation and RT-qPCR

Total RNA was extracted from the 24 mm inserts with the PureLinkTM RNA Mini Kit (Thermo Fisher Scientific). cDNA was synthesized from 500 ng of total RNA for each sample using the iScript^TM^ Reverse Transcription Supermix for RT-qPCR (Bio-Rad, Hercules, CA, USA). The mRNA level of gene expression was measured using iTaq Universal SYBR Green Supermix (Bio-Rad) in a QuantStudio 6 Flex qPCR machine (Thermo Fisher Scientific). The primers used in this study are IL1β (forward: CAA-CCA-ACA-AGT-GAT-ATT-CTC-CAT-G; reverse: GAT-CCA-CAC-TCT-CCA-GCT-GCA) [17], iNOS (forward: AAT-CTT-GGA-GCG-AGT-TGT-GG; reverse: CAG-GAA-GTA-GGT-GAG-GGC-TTG) [18], arginase 1 (Arg1) (forward: AAC-ACG-GCA-GTG-GCT-TTA-AC; reverse: GAG-GAG-AAG-GCG-TTT-GCT-TA) [17], and the housekeeping gene GAPDH (forward: GCA-CAG-TCA-AGG-CCG-AGA-AT; reverse: GCC-TTC-TCC-ATG-GTG-GTG-AA).

### 2.7. Bulk RNA Sequencing

The total RNA was also submitted for bulk RNA sequencing. RNA integrity was measured by the Agilent BioAnalyzer 2100 (Agilent Technologies, Santa Clara, CA, USA). The libraries were prepared and sequenced in single-read mode with 75 bases (75 SR). The read depth was 25–35 M reads per sample. Library preparation and sequencing were performed at the Next Generation Sequencing Core at UT Southwestern Medical Center. The differentially expressed genes (DEGs) were identified between Fat and Con. The DEG analysis was performed using edgeR Bioconductor v. 3.14 [19]. We also analyzed the top 500 upregulated and 500 downregulated DEGs ranked based on their *p*-values using the Metascape website [20], which integrates multiple databases to annotate genes and perform pathway enrichment and network analyses.

### 2.8. Statistical Analysis

GraphPad Prism v. 10.1.0 (GraphPad Software Inc., La Jolla, CA, USA) was applied for data visualization and statistical analysis. Data are presented as mean ± standard deviation (SD). One-way ANOVA with post hoc Tukey’s multiple-comparison test was used in this study. A *p*-value ≤ 0.05 was considered statistically significant.

## 3. Results

### 3.1. Phagocytosis of Fat by Macrophages in Human ONFH Sample

**Figure 1:**
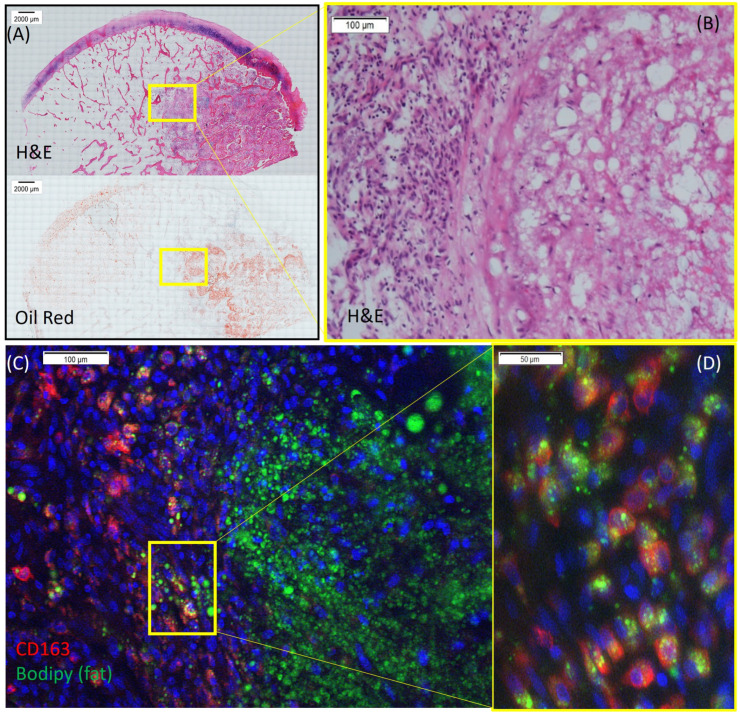
The phagocytosis of fat by macrophages was observed in human osteonecrosis of the femoral head (ONFH) in a patient sample. (**A**). Scans of H&E- and Oil Red O-stained human ONFH samples (4× scan). (**B**). A high magnification of the H&E-stained boundary area (10×). (**C**). The fat Bodipy™- and CD163-stained boundary area (10×). (**D**). A high magnification of fat Bodipy™ and CD163 double-positive cells (20×).

In ON, the reactive zone describes a histological region that lies between the healthy bone and the necrotic tissue and acts as an invasion front populated with various immune cells, bone progenitors, and endothelial cells. In the human ONFH specimen, we observed dense Oil Red O-positive fat staining in the necrotic area slowly penetrating the reactive zone (Figure 1A,B). Meanwhile, increased numbers of CD163-positive macrophages were observed in the reactive zone (invasion boundary area, Figure 1C). Interestingly, fat Bodipy™ staining co-localized within CD163-positive macrophages, indicating the active phagocytosis of necrotic fat by invading macrophages in this area (Figure 1D).

### 3.2. Phagocytosis of Fat by Macrophages in ON Mouse Model

**Figure 2:**
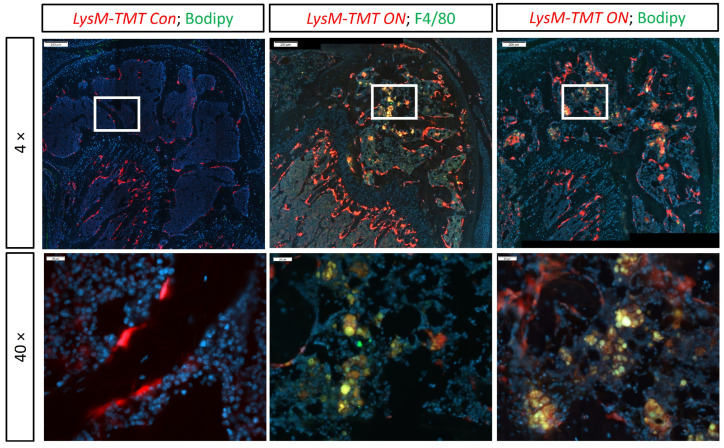
An F4/80- and Bodipy™-stained osteonecrotic epiphysis of the distal femur from LysM-TMT mice. Immunofluorescence staining shows LysM+ cells co-label with F4/80 and Bodify (fat) in the ON mouse model. (*n* = 3 animals).

The macrophage phagocytosis of necrotic fat was also observed at 4 weeks after ON induction in the ON mouse model. LysM-TMT mice expressed red fluorescence (tdTomato) in their myeloid cell lineage, including monocytes, mature macrophages, and osteoclasts. The co-labeling of tdTomato-positive cells and F4/80-positive IF-stained macrophages validated the mature macrophage labeling with tdTomato in the necrotic bone marrow (Figure 2). Similar to the human ONFH specimen, in the necrotic bone marrow of ON mice, green fat Bodipy™ staining co-localized within tdTomato-positive macrophages (Figure 2). We did not find any Bodipy™-stained adipocytes or lipid droplets in the contralateral control bone marrow, which is consistent with what we observed before in mice at this age (Figure 2). This observation confirmed the phagocytosis of necrotic fat by macrophages in the ON mouse model.

### 3.3. Macrophages and Necrotic Fat Co-Culture

**Figure 3:**
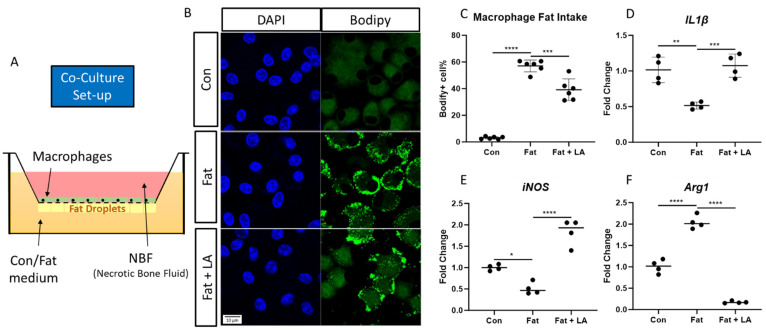
Macrophages and necrotic fat co-culture system. (**A**). The set-up of the co-culture system. (**B**). DAPI- and Bodipy™-stained cells in the co-culture system. (**C**). A comparison of the percentages of Bodify-positive cells among control, fat, and fat + LA (Latrunculin A) conditions. (**D**). A comparison of the expression of *IL1β* in cells among control, fat, and fat + LA conditions. (**E**). A comparison of the expression of *iNOS* in cells among control, fat, and fat + LA conditions. (**F**). A comparison of the expression of *Arg1* in cells among control, fat, and fat + LA conditions. (*: *p* ≤ 0.05, **: *p* ≤ 0.01, ***: *p* ≤ 0.001, ****: *p* ≤ 0.0001.)

To study the effects of fat phagocytosis on the macrophage phenotype, we set up an in vitro co-culture system in which transwell-seeded primary macrophages could make direct contact with fat (Figure 3A). We also provided aNBF in both the control fat- and high-fat-containing media to mimic the necrotic environment. We used Western blotting to validate the presence of DAMPs such as HMGB1, Cyclophilin A, and S100A6 in mouse aNBF (Figure S1). For the cells cultured in the fat control medium, we barely found any Bodipy^TM^-positive macrophages. However, for the cells cultured in the high-fat-containing medium, around 57% of macrophages were Bodipy^TM^-positive, which was significantly higher than in Con (*p* < 0.0001) (Figure 3B,C). These results confirmed necrotic fat phagocytosis by macrophages in this co-culture system. Additionally, fat phagocytosis significantly decreased the expression of M1 markers such as *IL1β* (*p* < 0.01) and *iNOS* (*p* < 0.05) in macrophages (Figure 3D,E), whereas the expression of the M2 marker *Arg1* was significantly increased with fat phagocytosis (*p* < 0.0001) (Figure 3F). To investigate whether the polarization change is indeed mediated by phagocytosis, we treated the cells with LA, which restrained the phagocytosis activities of macrophages. LA supplementation decreased the macrophage fat intake in the high-fat-containing condition, and the Bodipy^TM^-positive macrophages significantly decreased to around 39% (*p* < 0.001) (Figure 3B,C). Remarkably, LA reversed the polarization marker gene changes induced by fat phagocytosis: the expression of the M1 markers *IL1β* (*p* < 0.001) and *iNOS* (*p* < 0.0001) increased significantly, while the expression of the M2 marker *Arg1* (*p* < 0.0001) decreased (Figure 3D–F). This indicated that the phagocytosis of necrotic fat could induce the macrophages to polarize to an M2 anti-inflammatory type. In addition, the inhibition of phagocytosis could reverse this polarization change.

### 3.4. Effects of Fat Phagocytosis on Transcriptional Profile of Macrophages

**Figure 4:**
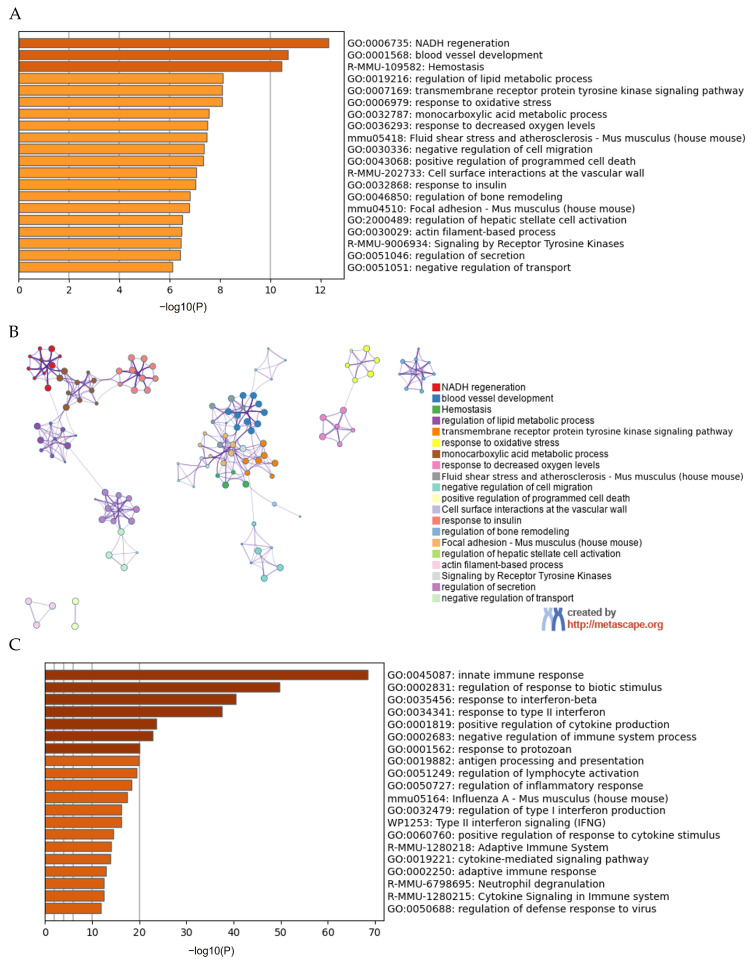

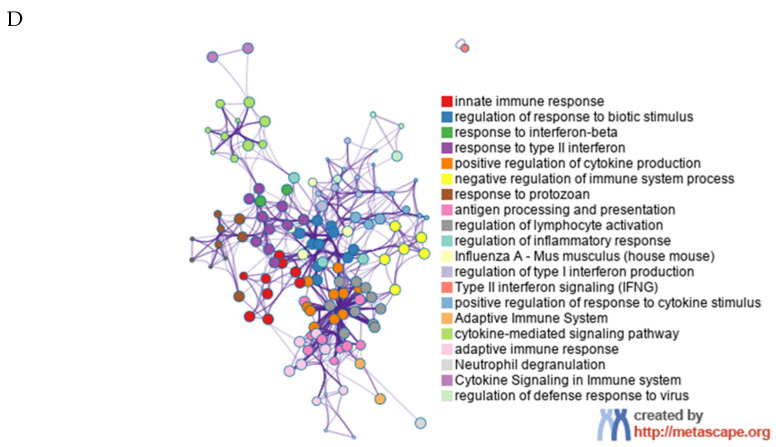
Pathway enrichment and network analyses of differentially expressed genes (DEGs). (**A**). A Metascape bar graph of the top 20 non-redundant enriched clusters from the top 500 upregulated DEGs. (**B**). Metascape enrichment network visualization of the top 20 non-redundant enriched clusters from the top 500 upregulated DEGs (colored by Cluster ID). (**C**). A Metascape bar graph of the top 20 non-redundant enriched clusters from the top 500 downregulated DEGs. (**D**). Metascape enrichment network visualization of the top 20 non-redundant enriched clusters from the top 500 downregulated DEGs (colored by Cluster ID).

The transcriptional profiles of macrophages cultured in the necrotic fat control medium and the necrotic high-fat-containing medium were analyzed after bulk RNA sequencing. DGE analysis revealed the top upregulated genes related to anti-inflammatory responses, like *Adipoq*, *Arg1*, and *DUSP* family genes (Supplementary Table S1). Proinflammatory genes such as *IL1β*, *TNF*, *TLR4*, the *CXCL* family, and the *CCL* family were significantly downregulated (Supplementary Table S2). Additionally, pathway enrichment and network analyses for the top 500 upregulated and 500 downregulated DEGs were performed. We found that anabolic signaling pathways such as blood vessel development, lipid homeostasis, response to oxidative stress, and regulation of bone remodeling were upregulated in macrophages with fat phagocytosis (Figure 4A,B). In comparison, enriched categories, including both innate and adaptive immune responses, multiple interferon subtypes, regulation of cytokine production, cytokine-mediated signaling pathways, and regulation of inflammatory response, were all significantly downregulated (Figure 4C). Interestingly, almost all top downregulated enriched clusters were linked together, sharing similar proinflammatory characteristics (Figure 4D). Therefore, the transcriptional findings clearly demonstrated the anti-inflammatory effects of fat phagocytosis. These unbiased RNA sequencing results further confirmed the impact of fat phagocytosis on macrophage polarization in the context of ON.

### 3.5. Changes in Macrophage Markers after ON In Vivo

**Figure 5:**
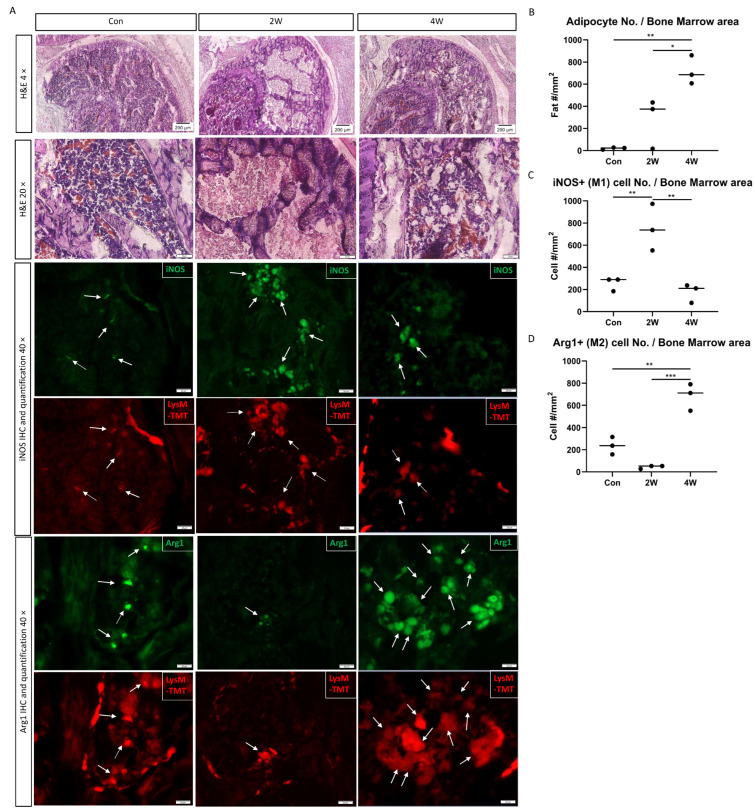
The changes in macrophage markers in vivo after ON. (**A**). A comparison of the H&E-, iNOS-, Arg1-, and LysM-labeled images of the osteonecrotic epiphysis of the distal femur in LysM-TMT mice among the control and 2 weeks and 4 weeks after ON induction. The white arrows point to the double positive cells of iNOS and LysM-TMT, or Arg1 and LysM-TMT. (**B**). A comparison of the adipocyte number in bone marrow among the control and 2 weeks and 4 weeks after ON induction. (**C**). A comparison of the iNOS-positive cells in bone marrow among the control and 2 weeks and 4 weeks after ON induction. (**D**). A comparison of Arg1-positive cells in bone marrow among the control and 2 weeks and 4 weeks after ON induction. (*: *p* ≤ 0.05, **: *p* ≤ 0.01, ***: *p* ≤ 0.001.)

The same macrophage phenotype changes induced by fat phagocytosis were also observed in vivo. We investigated ON mice at 2 weeks and 4 weeks after ON induction. The number of adipocytes increases with the progression and healing of ON. At 4 weeks after ON, the number of adipocytes in the necrotic bone marrow was significantly higher than that in the control (*p* < 0.01) and that at 2 weeks (*p* < 0.05) (Figure 5A,B). At 2 weeks after ON induction, most tdTomato-positive macrophages were co-labeled with iNOS rather than Arg1 via IF staining, and the number of iNOS-positive macrophages significantly increased compared to the control (*p* < 0.01) (Figure 5A,C). With increased marrow fat content and fat phagocytosis by macrophages at 4 weeks post-ON, most tdTomato-positive macrophages were co-labeled with Arg1 rather than iNOS by IF staining (Figure 5A). The number of iNOS-positive cells significantly decreased at 4 weeks compared to 2 weeks (*p* < 0.01) (Figure 5C), while Arg1-positive macrophages increased at 4 weeks when compared to 2 weeks after ON (*p* < 0.001) (Figure 5D).

## 4. Discussion

In the current study, we discovered that increased fat phagocytosis by macrophages is present in both human ON and the ON mouse model. Fat phagocytosis led to a decrease in *IL1β* and *Arg1*/*iNOS* RNA expression using an in vitro co-culture system, while using LA significantly reversed the macrophage gene changes by blocking phagocytosis, suggesting that fat phagocytosis in the necrotic environment promotes an anti-inflammatory phenotype switch in macrophages. This notion was further validated using the unbiased bulk RNA sequencing technique. GO and pathway analysis showed significantly decreased immune/inflammatory responses, alongside increased anabolic signaling pathways, such as vessel development, lipid homeostasis, and bone remodeling, indicating activated pro-tissue-healing cascades. In vivo analysis consistently showed a switch from a high-iNOS/Arg1 macrophage population in the early healing phase to a low-iNOS/Arg1 population at 4 weeks post-ON induction when fat phagocytosis is active. Our study is the first to demonstrate that fat phagocytosis promotes a macrophage phenotype switch from proinflammatory to anti-inflammatory in ON. We think that the findings of this study expand our knowledge of the function of macrophages in ON and point to new directions for ON treatment targeting osteoimmunology.

To mimic the osteonecrotic environment in vitro, we adopted a previously established aNBF treatment method [2]. This method is induced by cycles of freezing and thawing using fresh sterile bone, causing acute necrotic cell death similar to that in vivo. Acute cell death releases phenotypical DAMPs, including HMGB1, s100, and Cyclophilin A, and is demonstrated to consistently and reliably induce a necrotic cell response and stress in vitro. In a previous study [2], we also compared the content and cell response between aNBF and “true” NBF, which we are only capable of isolating from bones in large animals with ON due to the sample size. We showed that both aNBF and “true” NBF are comparable in their DAMP content and similarly skew MSC differentiation toward fibrogenesis rather than osteogenesis.

In ON, it has been widely noted that a sharp increase in marrow fat content is present in human patients and various animal models [9,10,21,22]. Marrow fat secretes several adipokines, such as Leptin, Adiponectin, TNFα, IL1β, and IL6, which promote chronic inflammatory responses and are shown to be highly correlated with bone cancer metastasis and markers for the progression of bone diseases such as osteoporosis [23,24]. In addition, marrow fat in the necrotic tissue may contain a wide spectrum of fatty acids, including oxidized lipids. We recently found that oxidative stress and oxidized LDL were significantly elevated in ON, which inhibited endothelial cell migration and angiogenesis in the HUVEC culture system. Consistently, our in vivo study suggested that increased marrow adiposity impairs revascularization and bone remodeling in an ON obese mouse model [10], potentially due to an aggravated oxidative stress burden and further elevated oxLDL levels. Interestingly, the current report provides a novel mechanism of macrophage polarization to a pro-tissue-healing phenotype via marrow fat phagocytosis. The authors believe that the increase in marrow fat content in ON may function as a double-edged sword. On the one hand, increased marrow fat attracts macrophages, intensifying the inflammatory response to resolve tissue damage, and promotes macrophage switching to an anti-inflammatory phenotype; on the other hand, if not promptly resolved, increased marrow fat can also lead to a chronic response via secreting inflammatory cytokines. The balance of the tissue response to marrow fat is critical in mediating anabolic responses of bone healing in ON.

Macrophages are plastic cells and may polarize to different phenotypes in response to environmental changes. It is well characterized that, in vitro, macrophages can be classified into M1 (classically activated) and M2 (alternatively activated) phenotypes due to their different responses to stimuli, such as LPS and IL4 [6,7]. INOS and Arg1 are the classic marker genes for in vitro M1 and M2 phenotypes, respectively, as it was demonstrated that arginine was competitively metabolized via different pathways in M1 vs. M2 [25]. In M1 macrophages, arginine is metabolized to NO and citrulline via nitric oxide synthase (NOS), whereas in M2, arginine is favored to be metabolized to ornithine and urea via arginase (ARG). In our in vitro necrotic fat co-culture experiment, we found a significant upregulation of Arg1 and downregulation of iNOS expression, suggesting that an M2-like phenotype switch is triggered by fat phagocytosis in macrophages. Phagocytosis of fat/lipids has also been indicated in other contexts, such as cancer, obesity, and atherosclerosis via a CD36-mediated mechanism [26,27,28]. It was shown that the choice between lipid oxidation and glycolysis plays a crucial role in macrophage polarization [29]. There is also an interesting observation on the ploidy of macrophages that are capable of fat phagocytosis. Some refer to these macrophages as adipoclasts [30]. In our study, we found that both single- and multi-nucleated macrophages are capable of fat phagocytosis in vivo and in vitro in the co-culture system, and there is no difference in their ability to perform fat phagocytosis based on Bodipy/Oil red O staining, although more advanced spatial sequencing techniques may be able to address their gene expression differences. Phagocytosis is a highly regulated cellular mechanism with the function of clearing cell debris and resolving inflammatory responses. Studies have shown that M2 macrophages have a high capacity for phagocytosis in mice in response to HSV infection [31]. An M2-like phenotype may be activated in ON by fat phagocytosis to further enhance the capability of macrophages to resolve inflammation and promote tissue healing. Nonetheless, we do acknowledge that the macrophage phenotypes are more complex in vivo and that the M1/M2 classification is inaccurate and an over-simplification of the phenotypes.

Macrophage activity and its phenotypes may directly affect osteoblast and osteoclast functions [32]. Osteoclasts derive from the monocyte–macrophage lineage. Studies have shown that an increased M1 phenotype is highly associated with osteoclast formation [33]. M1 macrophages secrete proinflammatory cytokines such as IL1β and TNFα, which showed a synergistic effect with RANKL in promoting osteoclast formation, whereas M2 macrophages inhibit osteoclast formation and have been shown to stimulate osteoblast proliferation and bone formation in a low-dose irradiation cranial defect rat model [34]. In our ON model, the fat-phagocytosis-mediated macrophage phenotype change is also in line with the healing responses. At 2 weeks post-ON induction, we observed a high-M1-like macrophage population when marrow fat content was low and bone formation was absent. In comparison, at 4 weeks post-ON, increased M2-like macrophages were present when marrow fat content increased and bone formation was active.

There are a few limitations in this study. First, the biological effects of fat phagocytosis in human ON were not studied. Although both mouse and human ON show similar phenotypes of fat phagocytosis in macrophages, mouse macrophage biology may not fully recapitulate human cell responses. Human macrophage responses are likely to be more complex, especially since sustained steroid use, a common cause of ON in humans, may influence macrophage behavior. Second, the fat phagocytosis phenotype was studied using an in vitro co-culture system. Future studies using spatial transcriptomics may be superior in defining the in vivo changes in macrophages with the correspondence of cells’ histological locations (as shown in Figure 1) during ON healing.

## 5. Conclusions

In the present study, we discovered that fat phagocytosis by macrophages occurs in both a human ON patient and a mouse model. Fat phagocytosis promotes an anti-inflammatory response in macrophages, and blocking it reverses these effects. The macrophage phenotype change also correlates with the in vivo healing responses observed in histology. Our study reveals a unique macrophage polarization mechanism triggered by phagocytosis in ON, suggesting potential new therapeutics targeting macrophage immunology in ON.

## Data Availability

RNA sequencing data are available via the GEO (Gene Expression Omnibus) repository (GEO accession number GSE272784).

## Supplementary Materials

The following supporting information can be downloaded at https://www.mdpi.com/article/10.3390/cells13141227/s1: Figure S1: The scheme of macrophage, necrotic bone fluid, fat control medium, and high-fat-containing medium acquisition from mice; Figure S2: Confocal images of stained mouse macrophage in the co-culture system; Table S1: Top 500 upregulated genes; Table S2: Top 500 downregulated genes.
