# Supplementary material for: Fat Phagocytosis Promotes Anti-Inflammatory Responses of Macrophages in a Mouse Model of Osteonecrosis
Source: Cells. 2024 Jul 20;13(14):1227. doi: 10.3390/cells13141227 (PMC11274809; doi:10.3390/cells13141227)
Supplement: Supplementary file 1 [file cells-13-01227-s001.zip › supplementary figures.pdf]

Supplementary Figure 1

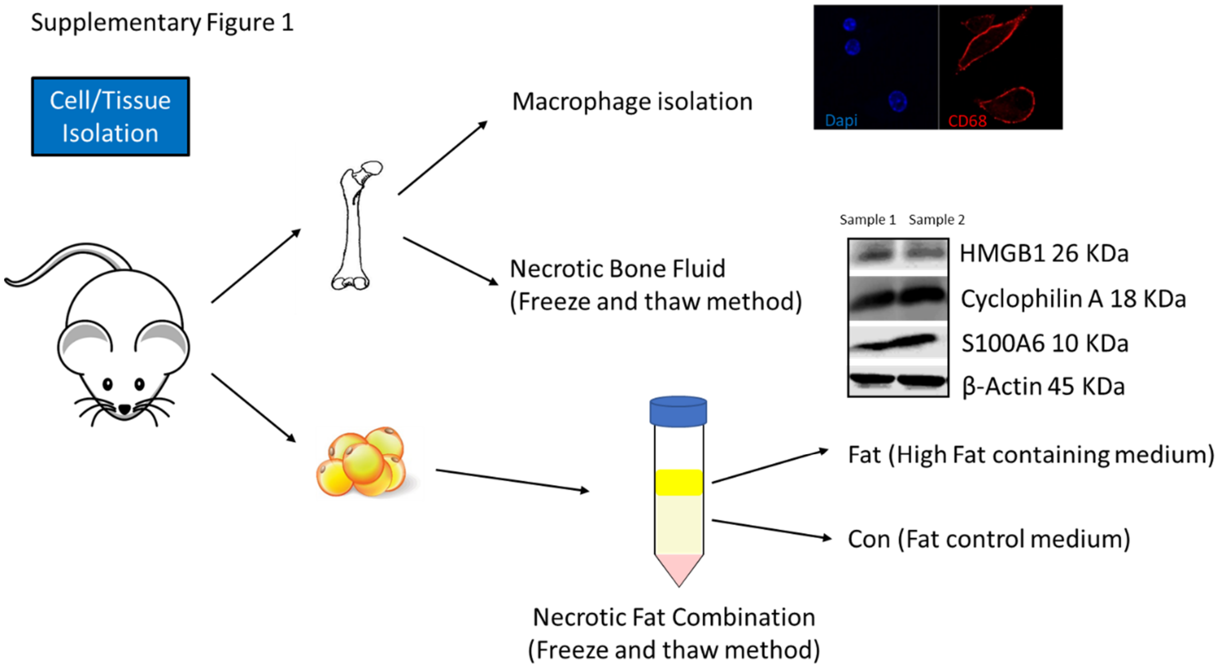

Supplementary Figure S1. The scheme of the macrophages, necrotic bone fluid, fat control medium, and high fat containing medium acquisition from mice. Western blot shows phenotypical DAMPS presence in mouse necrotic bone fluid such as HMGB1, Cyclophilin A and S100A6. Proteins for western are isolated in two different batches of experiments and showed high consistency in DAMPs expression.

Supplementary Figure 2

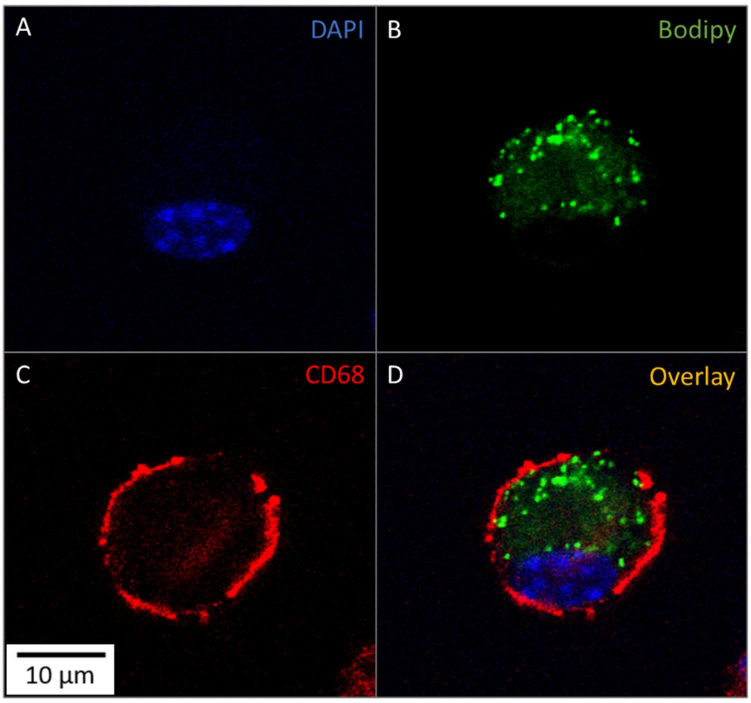

Supplementary Figure S2. Confocal images of stained mouse macrophage in the co-culture system. A). DAPI staining of the nuclei as blue. B). Bodipy™ staining of the fat droplets are as green. C). CD68 staining for the macrophage surface marker as red. D). Overlays of all three colors. This picture confirmed the phagocytosis of fat droplets are inside of the cell membrane.
